# Clinical Validation of the Champagne Algorithm for Evoked Response Source Localization in Magnetoencephalography

**DOI:** 10.1007/s10548-021-00850-4

**Published:** 2021-06-11

**Authors:** Abhishek S. Bhutada, Chang Cai, Danielle Mizuiri, Anne Findlay, Jessie Chen, Ashley Tay, Heidi E. Kirsch, Srikantan S. Nagarajan

**Affiliations:** 1 Biomagnetic Imaging Laboratory, Department of Radiology and Biomedical Imaging, UCSF Biomagnetic Imaging Center, 513 Parnassus Avenue, San Francisco, CA 94143, USA; 2 Department of Neurology, Epilepsy Center, UCSF, 94143 San Francisco, CA, USA

**Keywords:** MEG, Magnetoencephalography, MSI, magnetic source imaging, Brain mapping, Sensorimotor cortex, Functional mapping

## Abstract

Magnetoencephalography (MEG) is a robust method for non-invasive functional brain mapping of sensory cortices due to its exceptional spatial and temporal resolution. The clinical standard for MEG source localization of functional landmarks from sensory evoked responses is the equivalent current dipole (ECD) localization algorithm, known to be sensitive to initialization, noise, and manual choice of the number of dipoles. Recently many automated and robust algorithms have been developed, including the Champagne algorithm, an empirical Bayesian algorithm, with powerful abilities for MEG source reconstruction and time course estimation (Wipf et al. 2010; Owen et al. 2012). Here, we evaluate automated Champagne performance in a clinical population of tumor patients where there was minimal failure in localizing sensory evoked responses using the clinical standard, ECD localization algorithm. MEG data of auditory evoked potentials and somatosensory evoked potentials from 21 brain tumor patients were analyzed using Champagne, and these results were compared with equivalent current dipole (ECD) fit. Across both somatosensory and auditory evoked field localization, we found there was a strong agreement between Champagne and ECD localizations in all cases. Given resolution of 8mm voxel size, peak source localizations from Champagne were below 10mm of ECD peak source localization. The Champagne algorithm provides a robust and automated alternative to manual ECD fits for clinical localization of sensory evoked potentials and can contribute to improved clinical MEG data processing workflows.

## Introduction

Planning resective brain surgery, whether for the removal of structural lesion or of seizure onset zone, requires the estimation of the location of functional regions of cortex in order to plan a strategy that maximizes benefit while minimizing risk of postsurgical functional deficit. Magnetoencephalography (MEG) is particularly well-suited for functional preoperative brain mapping prior to surgery of tumor or vascular lesion because its results do not depend primarily on blood flow that can be altered by tumor growth and does not suffer from susceptibility artifacts and vascular confounds seen, e.g., with fMRI (Kreidenhuber et al. 2019). Like other neurophysiologic methods, it has exquisite temporal resolution, and with the choice and application of appropriate source imaging techniques, can provide results with a high degree of spatial resolution.

The clinical standard for MEG source localization of interictal epileptiform discharges (IEDs, or “spikes”) as well as for functional landmarks from sensory evoked responses is the equivalent current dipole (ECD) localization algorithm (Burgess et al. 2011). Despite its widespread use, the technique of manual ECD fitting is subjective, labor-intensive, and sensitive to noise. Many semi- and fully automated algorithms have been elaborated to counter these difficulties. These have included, for example, the application of the coherent Maximum Entropy of the Mean (cMEM) approach as described in Chowdhury et al. (2013) to the localization of IEDs, a method termed distributed magnetic source imaging or dMSI, and found to be successful in comparison to ECD fitting by Pelligrino et al. (2018). Recently, the Bayesian multidipole iterative Monte Carlo approach “SESAME” described by Sommariva and Sorrentino (2014) was evaluated in comparison to standard ECD methods by Luria et al. (2020) for the localization of IEDs, likewise showing excellent performance. These methods were also noted to be advantageous because results were more operator-independent.

Over the last decade we have developed and gained experience with an empirical Bayesian algorithm with powerful abilities for MEG source reconstruction and time course estimation (Wipf et al. 2010; Owen et al. 2012); in its current form, it is called Champagne. Here, we evaluate the performance of the Champagne algorithm in preoperative brain tumor patients for localization of functional cortices (sensory and auditory) where there was minimal failure in localizing sensory evoked responses using standard ECD methods. Our aim is to establish equivalence between the clinical standard of manual ECD fitting and automated Champagne in this scenario, and also to illustrate several cases where Champagne may provide specific benefit in the case of ECD failure.

## Clinical Materials and Methods

### Patient Characteristics

A retrospective analysis of our clinical MEG database identified 21 patients who underwent preoperative mapping of auditory and somatosensory cortices with magnetic source imaging between March 2018 and May 2018 before tumor resection at the University of California, San Francisco. Please refer to Table 1 for the demographic, clinical, and pathological characteristics of each patient. The final cohort consisted of 13 females and 8 males. The patients’ ages ranged from 26 to 69 years (mean age 49.3 ± 15.3 years). The patients self-reported their dominant hand; 17 patients (81 %) were right-handed and 4 (19 %) were left-handed (19.0 %). In order to compare results of Champagne and ECD fit analyses, we included only patients whose data were of sufficient quality to allow ECD analysis.

### Preoperative Clinical Neuroimaging

#### MEG

##### Data Acquisition

Magnetic fields were recorded in a shielded room using a whole-head MEG system (CTF Omega 2000, CTF MEG, Coquitlam, BC, Canada) consisting of 275 axial gradiometers and 29 reference sensors used for computing synthetic third-order gradiometer measurements. MEG signals were digitized at a sampling rate of 1200 Hz.

##### Tasks

During the MEG procedure, each individual lay supine on a comfortable bed. For the auditory task, a 1 kHz tone was presented binaurally for 400-msec duration with an interstimulus interval of 2000-msec and a jitter between 0 to 100-msec at random for a total of 120 trials. Data was collected in epochs of 600-msec with a 100-msec pre-stimulus interval for each trial. For the somatosensory task, lip and index fingers were painlessly stimulated using pneumatically driven pulses (20–25 psi, 40 msec duration) from balloon diaphragms. Epochs of 400-msec duration with a 100-msec pre-stimulus interval were collected for 512 trials, comprising 256 trials at each stimulus site: right and left lip sites (RLip, LLip) and right and left second digit (RD2, LD2). 512 trials of stimulus with an inter-stimulus interval of 500-msec and a jitter of 50-msec were randomly presented in one block. Two somatosensory blocks were performed in order to test a total of four somatosensory sites (RD2, RLip, LD2, and LLip). The location of the patient’s head position relative to the MEG sensors was determined at the beginning and ending of the collection by means of three small fiducial marks placed at nasion, left preauricular, and right preauricular points. The pre- and post-trial locations were used to measure head movement during each of the tasks (Kirsch et al. 2007; Nagarajan et al. 2008; Traut et al. 2019).

### Analysis of MEG Data

#### MEG Preprocessing

MEG sensor data for each task were averaged across trials after removing any trials with eye blink, muscle activity, or other obvious artifact. As a result, each subject had one averaged auditory evoked field dataset and four averaged somatosensory evoked field datasets (RD2, RLip, LD2, LLip). All MEG datasets were 3rd order gradient denoised and detrended. Next, the clinical MR images from a 3-tesla unit (GE Medical Systems) were transferred to MEG workstations and used in conjunction with MEG localization information for coregistration. Coregistration was done using CTF software (CTF Omega 2000, CTF MEG, Coquitlam, BC, Canada) and coregistration errors passed a maximum threshold of 0.5 cm.

#### Equivalent Current Dipole

The equivalent current dipole localization algorithm utilizes an iterative least squares minimization to compute the strength and location of dipoles in a single spherical volume of uniform conductivity that could account for the sensory data. The auditory evoked field data were filtered with a high pass between 1 and 4 Hz and for most cases filtered with a low pass of 40 Hz. The somatosensory evoked field data were filtered with a high pass between 1 and 15 Hz and for most cases filtered with a low pass of 40 Hz. The primary auditory cortex in each hemisphere was localized based on the M100 auditory response peak using ECD fit (Pross et al. 2015; Chang et al. 2016). The primary somatosensory cortex corresponding to each of the tactile stimulation sites was localized based on the earliest response peak ~ 45 msec post-stimulus onset seen in the contralateral hemisphere. Dipole fits were accepted based on a goodness-of-fit values > 0.95 and having 95 % confidence volume of reconstructions < 0.1 cm^3^.

#### Champagne Source Reconstruction

The Champagne algorithm is a robust empirical Bayesian method to estimate brain source localization and the time course of multiple neural sources of MEG data (Wipf et al. 2010; Owen et al. 2012). To apply Champagne, first the clinical MR images were spatially normalized to the Montreal Neurological Institute (MNI) atlas template using SPM (http://www.fil.ion.ucl.ac.uk/spm). Next, we calculated a three-component lead field for each voxel (e.g. see Huang et al. 1999) using a forward model of sensor activity at a spatial resolution of 8mm spanning the entire brain by using the NUTMEG open-source analysis toolbox in MATLAB (Dalal et al. 2008; Dalal et al. 2011; Owen et al. 2012). We used the NUTMEG software for visualization of source time-course, overlaid on coregistered MRIs. The time-series and lead-field calculations were estimated for voxels in the individual MRI space (co-registered to the MEG data) and then transformed to MNI coordinates. Spatiotemporal neural activity from both hemispheres following stimulus presentation was reconstructed. The activation patterns were then displayed on the MNI brain template for each subject.

In order to determine activation patterns, Champagne was deployed on every averaged dataset across each subject. The input to the automated Champagne algorithm were: (1) the MEG data, selected for the active time-window of interest; (2) the lead field matrix calculated from the forward model; and (3) baseline MEG data (pre-stimulus) used to calculate the model noise covariance. The only free parameter was the number of iterations used for the algorithm convergence, typically set to 100, based on experience with both simulations and with many real datasets. The algorithm output is the time course of source activity for each orientation, and source variance (power) for each voxel.

For comparison with ECD, we recorded the maximum of the estimated source activity within the active time window of interest. For the auditory evoked fields, we used a baseline time-window of − 100 to −5 ms pre-stimulus onset and an active time-window of 25–250 ms post-stimulus onset.For the somatosensory evoked fields, we used a baseline time-window of − 100 to − 5 ms pre-stimulus onset and an active time-window of 5–105 ms post-stimulus onset.

#### Comparison of Results Across Algorithms

Both the Champagne peaks and ECD localizations were chosen based on waveforms at consistent latencies yielding successful source localization results in the perirolandic region of the brain for the somatosensory evoked responses and in the auditory cortex for the auditory evoked responses. If a peak or dipole could not be found in these regions it was considered a failed localization. We calculated the success rate of each pipeline for each task across the cohort. Here, the success rate is defined as the number of successfully localized sources as described above out of the number of potential localizable sources for the auditory and somatosensory tasks across the cohort using each analysis.

For each stimulus, tri-planar MNI coordinates of the corresponding dipole fit and of the corresponding Champagne peak were recorded. As expected, the auditory task resulted in two sources from each hemisphere, while the somatosensory task resulted in a single source in the hemisphere contralateral to the stimulus presentation. Ideally, datasets for each subject thus included 12 total MRI coordinates, one from ECD fit and one from Champagne, for each stimulus: right AEF, left AEF, RD2, LD2, RLip, and LLip. Next, the Euclidean distances were calculated between ECD-Champagne coordinate pairs for each stimulus across subjects. The effectiveness of the Champagne algorithm in localizing source activity in this clinical population was determined based on how close the Champagne peaks were to the corresponding dipole fits for each stimulus. The cases in which a dipole location or Champagne peak could not be found were excluded from this analysis.

## Results

The overall success rate for source localization was greater for Champagne (96.8 %) than for ECD analysis (93.7 %). Table 2 provides a breakdown of the success rate of source localization for each stimulus type and analysis method across the cohort.

**Fig. 1:**
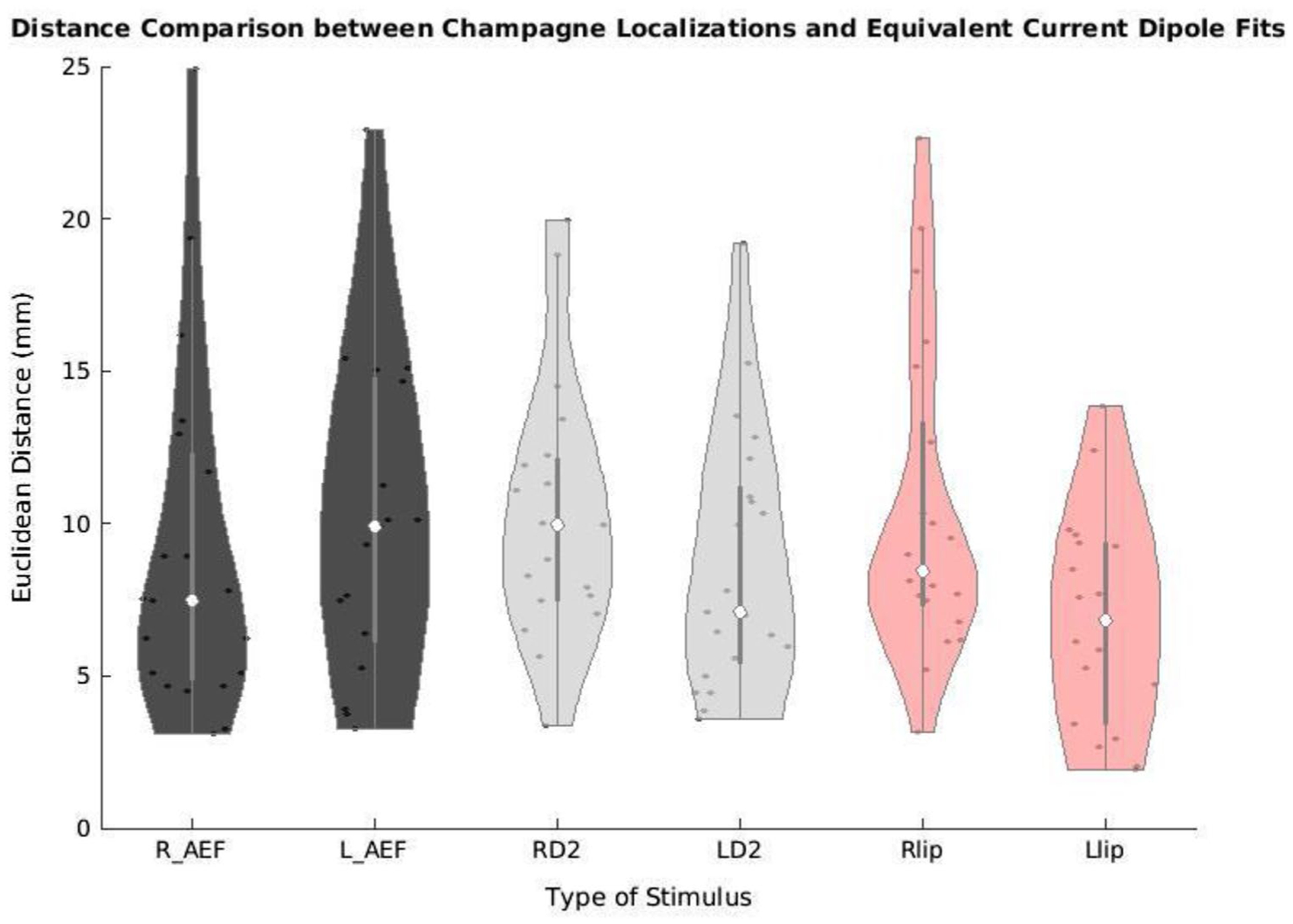
Distance between Champagne and ECD localization. Violin plot showing the difference in millimeters (y-axis) between Champagne peak activity and ECD locations for each stimulus type (x-axis). *R_AEF* right auditory evoked field; *L_AEF* left auditory evoked field; *RD2* right second digit somatosensory evoked field; *LD2* left second digit somatosensory evoked field; *RLip* right lip somatosensory evoked field; *LLip* left lip somatosensory evoked field

Across stimulus types and modalities, the average distance between Champagne peak and ECD location was 9.3 ± 4.8 mm. Figure 1 a violin plot showing the distances between Champagne peaks and ECD locations across each stimulus, provides a more detailed breakdown of the performance of Champagne analysis compared to ECD analysis for each category. Across the entire cohort, the average distance between Champagne peak and ECD location was 9.1 ± 5.7 mm for the right auditory evoked field and 10.1 ± 5.3 mm for the left auditory evoked field. The average distance between Champagne peak and ECD location was 11.9 ± 8.4 mm for the right index (RD2) somatosensory evoked field and 8.5 ± 4.1 mm for the left index (LD2) somatosensory evoked field. The average distance between Champagne peak and ECD location was 10.7 ± 5.7 mm for the right lip (RLip) somatosensory evoked field and 7.1 ± 3.3 mm for the left lip (LLip) somatosensory evoked field task. Overall, across these various stimulus types and modalities, the average distance between Champagne peak and ECD location was 9.3 ± 4.8 mm.

**Fig. 2:**
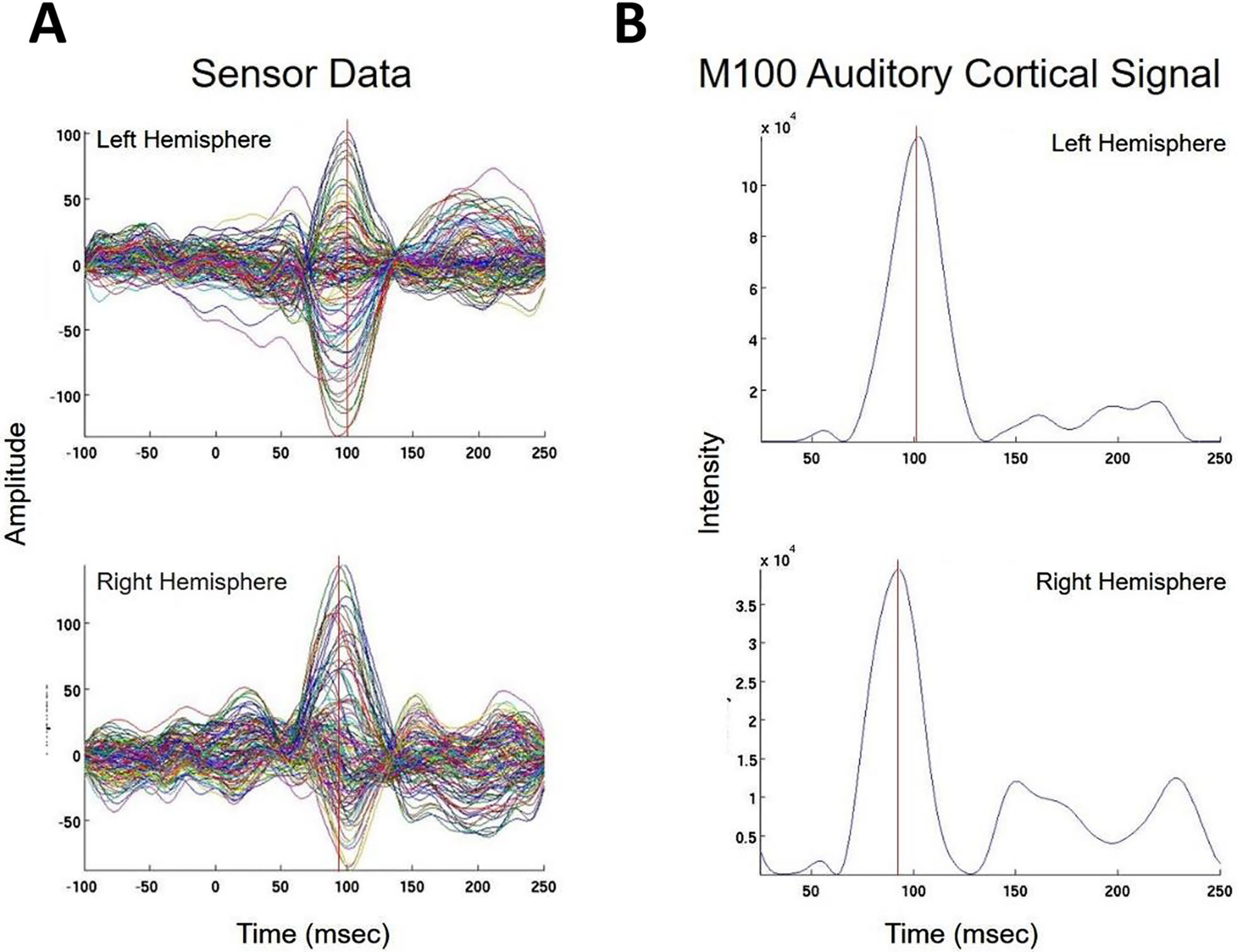

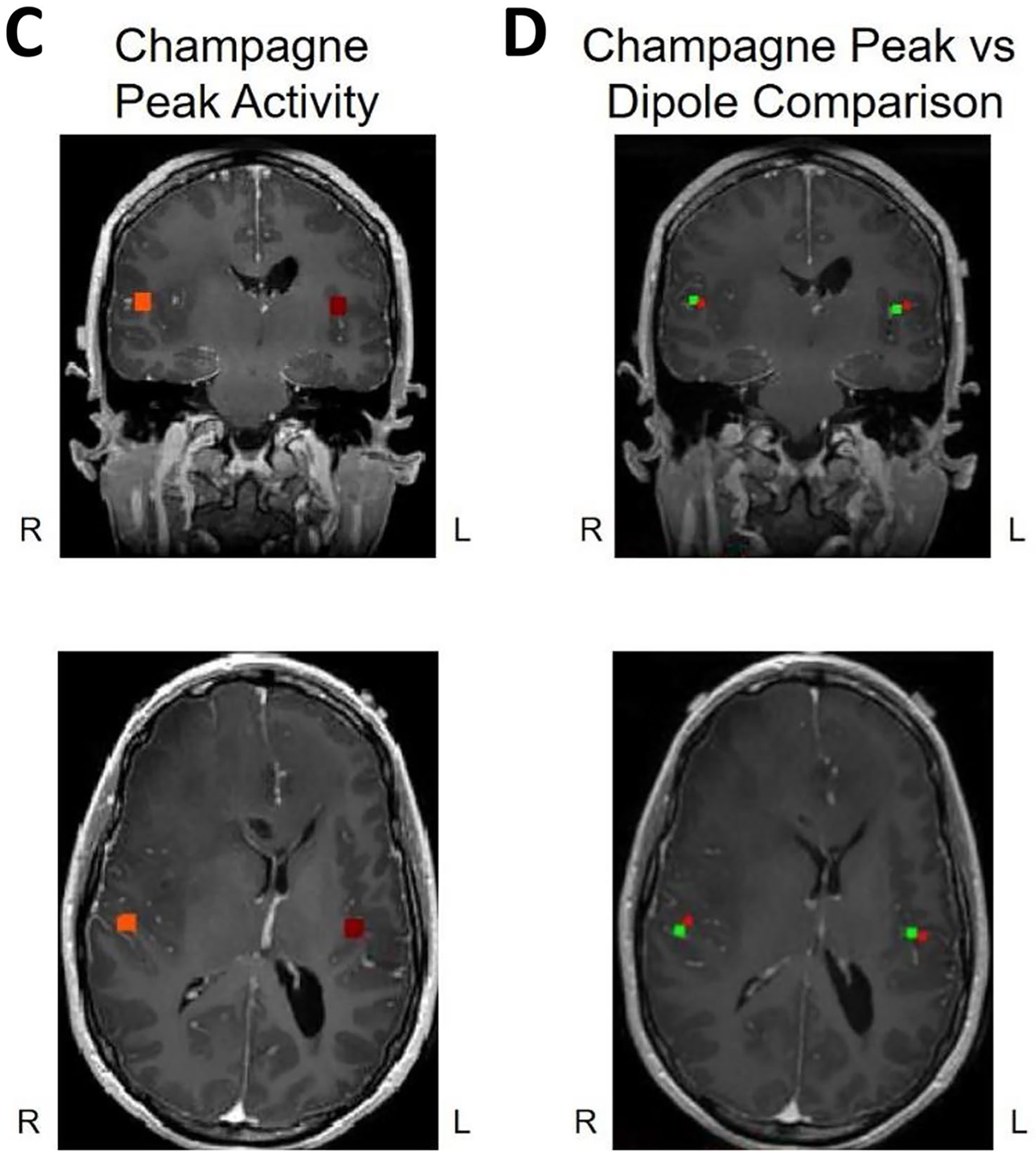
Concordant Auditory Evoked Field localization. AEF results for Patient 14 (concordant Champagne and ECD). Column A shows the time series of each sensor’s activity across time (msec). The top graph in column A overlays all left hemisphere sensors and the bottom graph in column A overlays all right hemisphere sensors. Column B displays the time course of cortical activity at the specific voxel corresponding to peak auditory cortical signal in the left (top) and right (bottom) hemispheres. The peak activity is at roughly 100 msec, consistent with the M100 auditory cortical response. Column C shows snapshots of brain MR slices in coronal and axial planes with the Champagne peak activity shown in red, corresponding to the peaks marked with red vertical cursors in column B. Column D overlays both Champagne peak (red circles) and ECD fit (green squares) on brain MR slices in coronal and axial plane

**Fig. 3:**
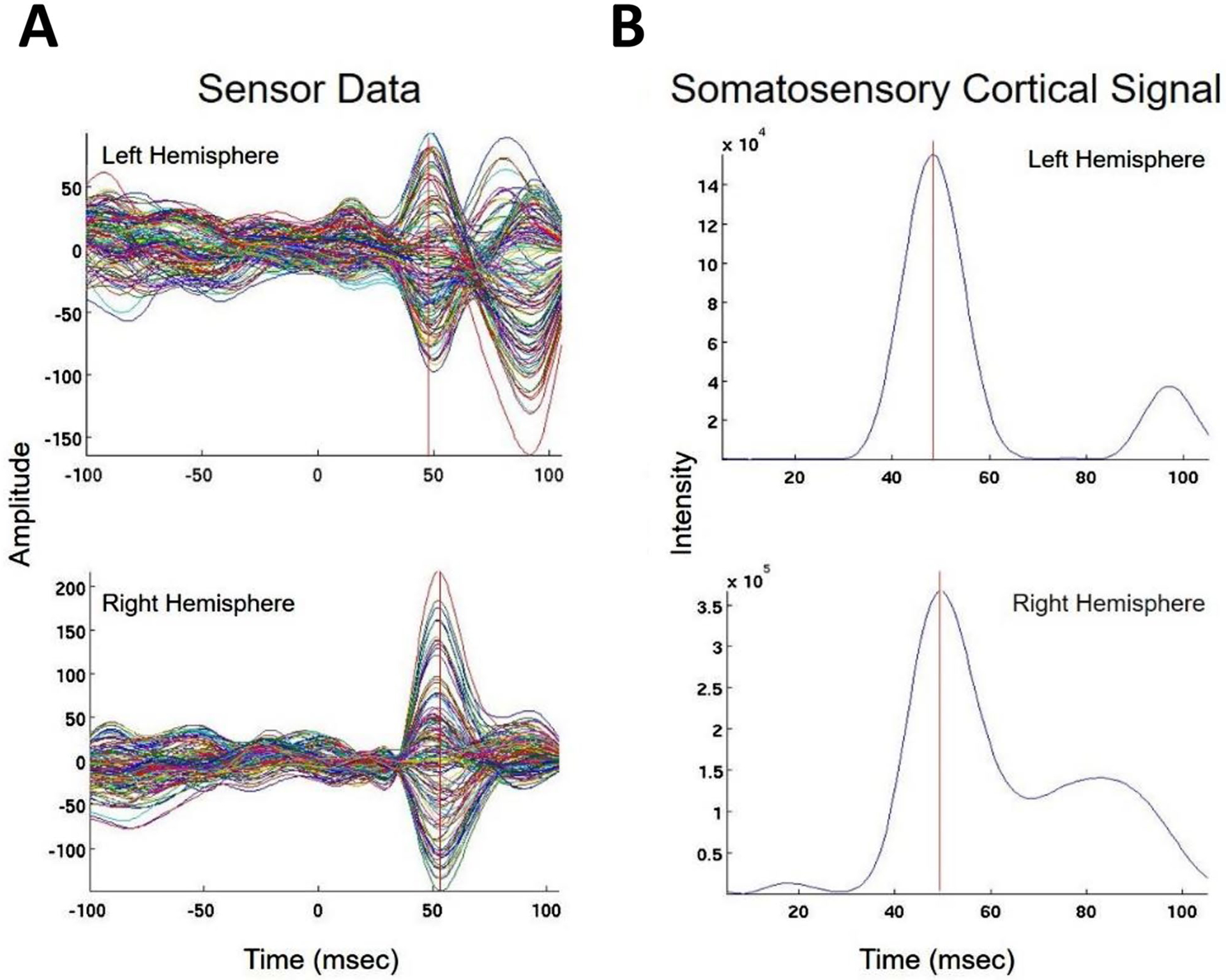

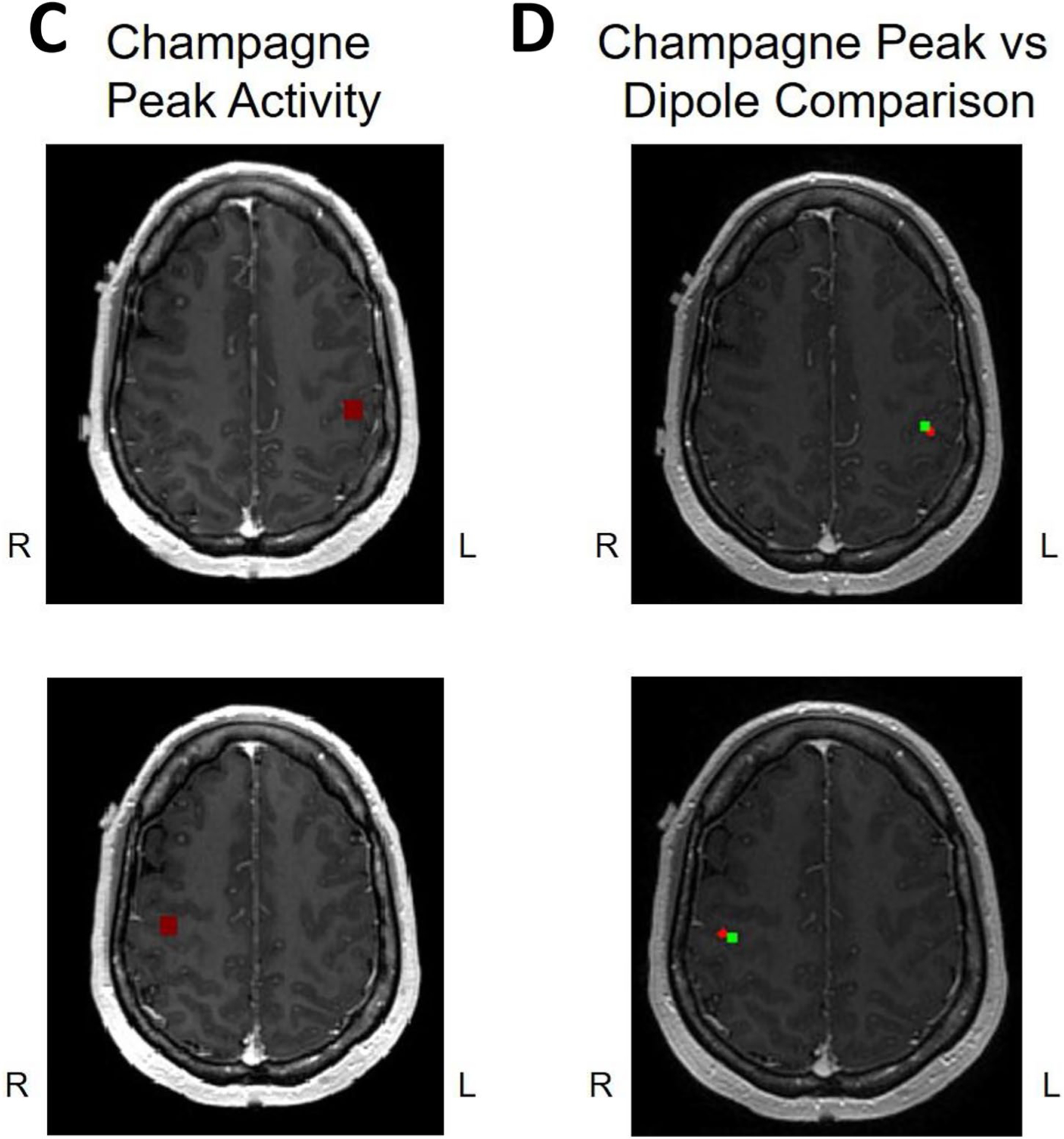
Concordant Index Finger (D2) Somatosensory Evoked Field localization. SEF D2 results for Patient 13 (concordant Champagne and ECD). Column A shows the time series of each sensor’s activity across time (ms). The top graph in column A overlays all left hemisphere sensors and the bottom graph in column A overlays all right hemisphere sensors. Column B displays the time course of cortical activity at the specific voxel corresponding to peak auditory cortical signal in the left (top) and right (bottom) hemispheres. The peak activity is between 53 and 57 ms, consistent with an index finger somatosensory cortical response. Column C shows snapshots of brain MR slices in the axial plane with the Champagne peak activity shown in red, corresponding to the peaks marked with red vertical cursors in column B. Column D overlays both Champagne peak (red circles) and ECD fit (green squares) on brain MR slices in coronal and axial plane

**Fig. 4:**
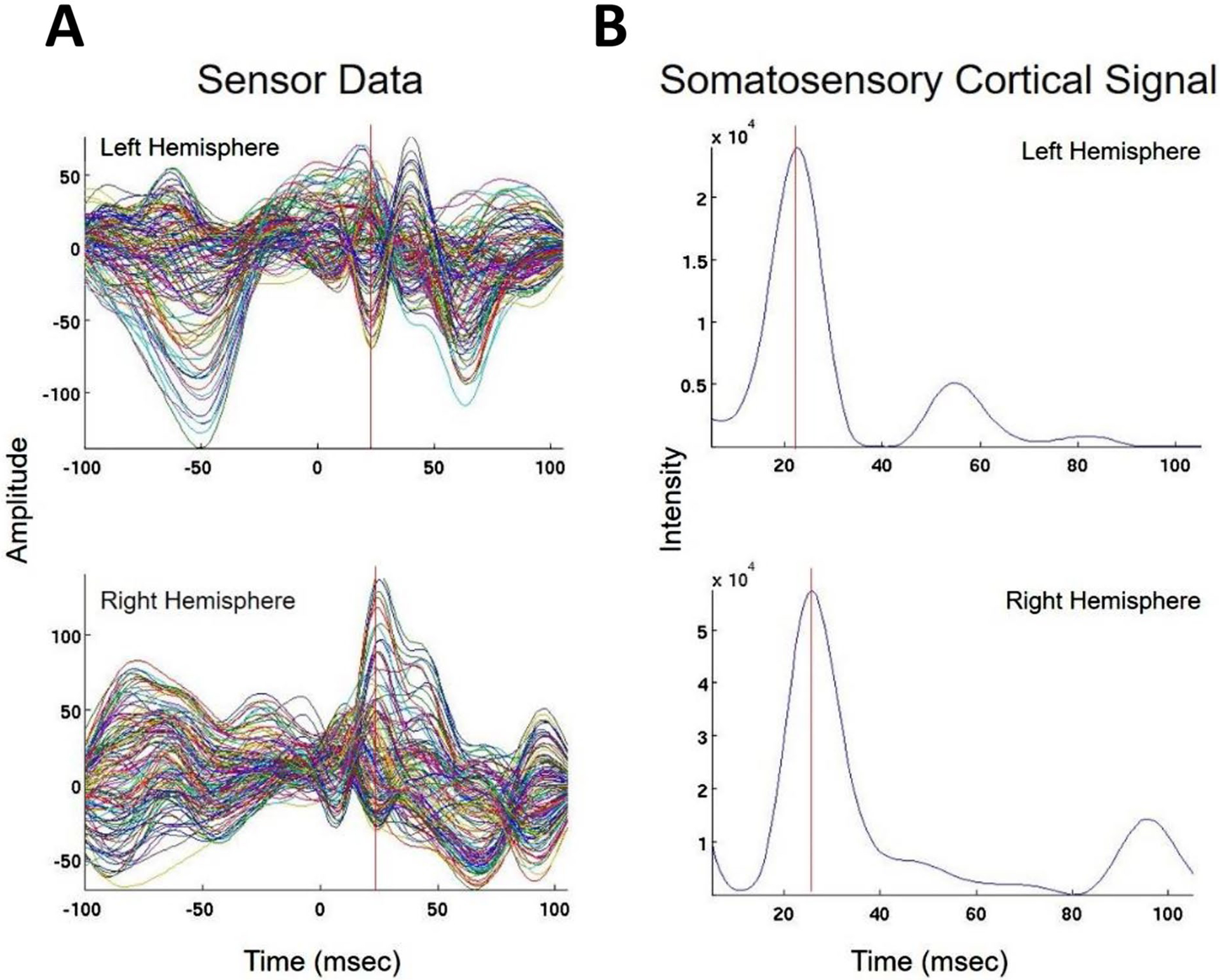

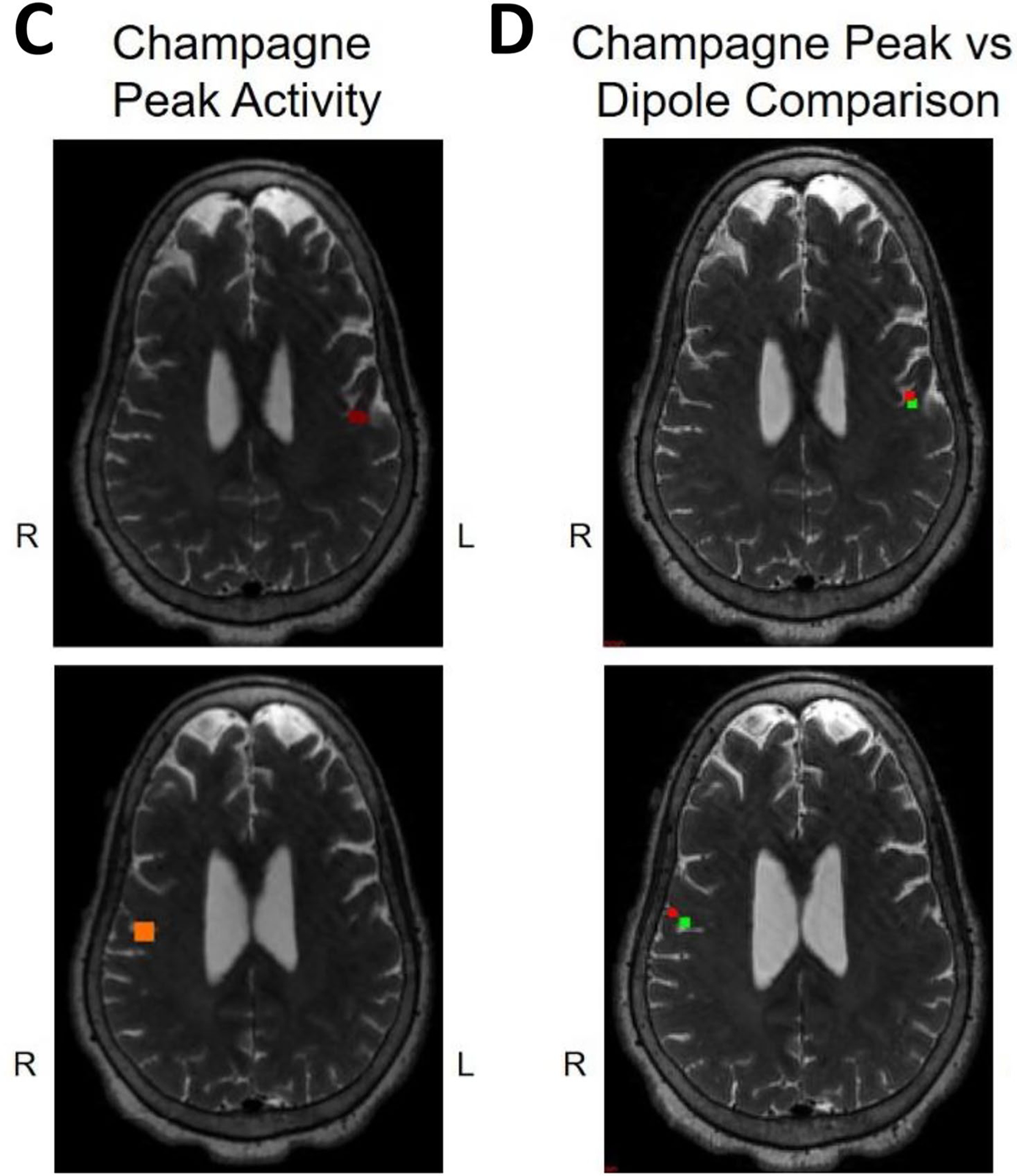
Concordant lip somatosensory evoked field localization. SEF Lip results for Patient 2 (concordant Champagne and ECD). Column A shows the time series of each sensor’s activity across time (msec). The top graph in column A overlays all left hemisphere sensors and the bottom graph in column A overlays all right hemisphere sensors. Column B displays the time course of cortical activity at the specific voxel corresponding to peak somatosensory cortical signal in the left (top) and right (bottom) hemispheres. The peak activity is between 21 and 23 msec, consistent with a lip somatosensory cortical response. Column C shows snapshots of brain MR slices in the axial plane with the Champagne peak activity shown in red, corresponding to the peaks marked with red vertical cursors in column B. Column D overlays both Champagne peak (red circles) and ECD fit (green squares) on brain MR slices in coronal and axial plane

We present cases that had high concordance between locations of Champagne peaks and respective ECD locations for each category of stimulus. These cases are shown in Figs. 2 and 3, and 4. Figure 2 is for Patient 14 who had concordant Champagne and ECD localization of auditory evoked fields (AEFs). Figure 3, for Patient 13, shows index finger somatosensory evoked field (SEFs) (top row RD2 stimulus and bottom row LD2 stimulus). Figure 4, for Patient 2, shows concordance between Champagne and ECD fit for lip SEFs (top row right lip stimulus and bottom row left lip stimulus); note that Champagne was successful in spite of a relatively noisy background in baseline signal.

## Discussion

We compared the performance of Champagne algorithm to the clinical standard for MEG source localization of sensory evoked fields, equivalent current dipole (ECD) fit. The Champagne peaks were on average < 10 mm away from corresponding ECD localization. We found strong concordance between Champagne and ECD for both auditory and somatosensory tasks throughout our clinical cohort.

Despite the success of Champagne algorithm, there are certain drawbacks that we predict might result in algorithm failure. Specifically, it is possible that the noise is not adequately modelled in the baseline due to artifactual data collection. In addition, there may be large co-registration errors that may result in faulty localization. We have circumvented such problems through standard troubleshooting steps that mitigate the effects of such issues.

In a few of the cases, Champagne was successful in localizing a source despite ECD failing to localize a source: this was true for three left and one right auditory evoked responses, and two left lip somatosensory responses. This provided insight into Champagne’s ability to provide localization even when ECD was unable to perform and provided some preliminary indication that Champagne algorithm was more robust than ECD fit in some cases. Further testing is still required to determine the sensitivity and specificity of Champagne against direct electrocortical stimulation mapping, a validated functional localization method that is considered to be clinical gold standard.

A tool like Champagne could also be especially useful in improving efficiency in MEG clinical data processing workflows. Current data processing workflows, especially with presurgical mapping data from brain tumor patients, can be taxing, labor intensive, and sensitive to error. Due to its automated procedures, we hope that the Champagne algorithm can be a useful and effective resource for source for a wide range of users. For example, since Champagne is an automated tool, it can be used as a first-pass source localization of sensory evoked fields. Champagne algorithm can also provide localization where ECD fitting is difficult or where it fails due to complexity of source activity, noisy data, or error of model. As noted above, as other groups have found for IEDs, algorithms that are robust in the face of technical limitations and that are usable for a wider range of users can provide practical solutions to clinical MEG source localization.

## Conclusion

In this study, we examine Champagne’s performance for source localization of sensory evoked fields in a clinical cohort. We provide strong evidence that the Champagne algorithm is equivalent to ECD fit. We also highlight Champagne as a viable alternative to the commonly clinically used ECD algorithm.

## Figures and Tables

**Table 1:** Clinical data in 21 patients who underwent MEG for localization of AEF and SEF

Case No.	Age (yrs), Sex	Handedness	Tumor type^a^	Tumor Location
1	27, F	R	Glioblastoma Grade IV	Right frontal
2	58, F	L	Low-grade astrocytic neoplasm	Left temporal
3	41, M	R	Neurenteric cyst	Right frontal
4	62, F	R	Anaplastic oligodendroglioma Grade III	Left insular
5	67, F	R	Glioblastoma Grade IV	Left parietal
6	66, F	R	Epithelioid glioblastoma vs. anaplastic epithelioid PXA	Left frontal
7	26, M	R	Diffuse astrocytoma Grade II	Left frontal
8	44, F	R	Diffuse glioma Grade II	Left frontal
9	63, F	R	High grade diffuse glioma Grade III	Right frontal
10	37, F	R	Anaplastic astrocytoma Grade III	Right frontal
11	66, M	R	Glioblastoma Grade IV	Left frontal
12	47, F	R	Anaplastic ependymoma Grade III	Left temporal
13	33, M	R	Glioblastoma Grade IV	Leftfrontal
14	43, F	L	Anaplastic astrocytoma Grade III	Right frontal
15	51, M	R	Glioblastoma Grade IV	Left temporal
16	31, F	R	Diffuse astrocytoma Grade II	Right frontal & left temporal lesions (preop for right frontal resection)
17	68, F	R	Low-grade glioma	Left temporal
18	26, M	L	Anaplastic oligodendroglioma Grade III	Left frontal
19	63, F	L	Meningioma Grade I	Right frontal
20	48, M	R	Glioblastoma Grade IV	Left frontal
21	69, M	R	Gliosarcoma	Right temporal

a Graded according to the World Health Organization system

**Table 2:** Success rate of Champagne and ECD for each stimulus type

Success rate	Champagne	ECD
AEF		
Right	100 % (21/21)	95.2 % (20/21)
Left	95.2 % (20/21)	81.0 % (17/21)
Combined	97.6 % (41/42)	88.1 % (37/42)
D2		
Right	90.5 % (19/21)	100 % (21/21)
Left	100 % (21/21)	100 % (21/21)
Combined	95.2 % (20/21)	100 % (21/21)
Lip		
Right	100 % (21/21)	100 % (21/21)
Left	95.2 % (20/21)	85.7 % (18/21)
Combined	97.6 % (41/42)	92.9 % (39/42)
Overall	96.8 %	93.7 %
